# Supplementary figures and images for: Metabolic and neurobehavioral disturbances induced by purine recycling deficiency in Drosophila
Source: eLife. 2024 May 3;12:RP88510. doi: 10.7554/eLife.88510 (PMC11068357; doi:10.7554/eLife.88510)

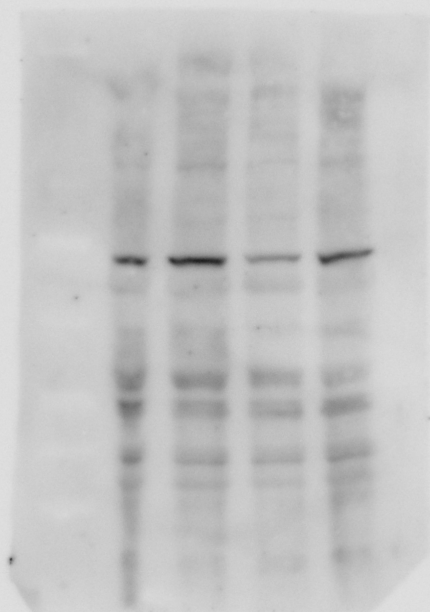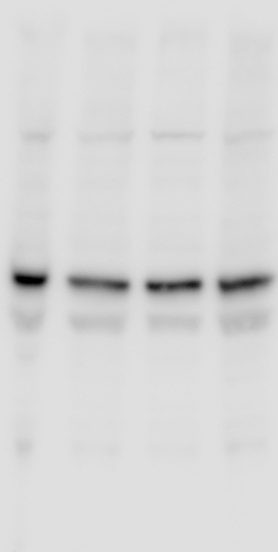

Supplement: Figure 5—source data 2. [file elife-88510-fig5-data2.zip › Figure 5 - source data 2.pdf]

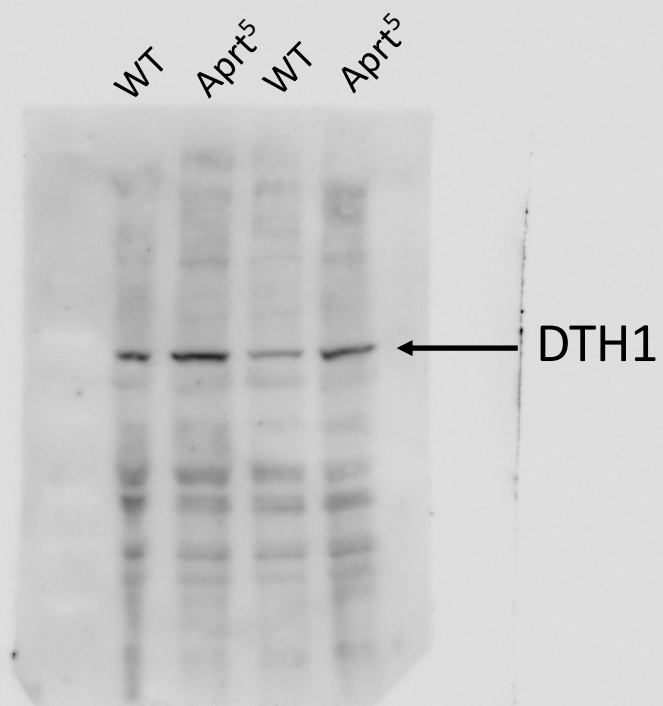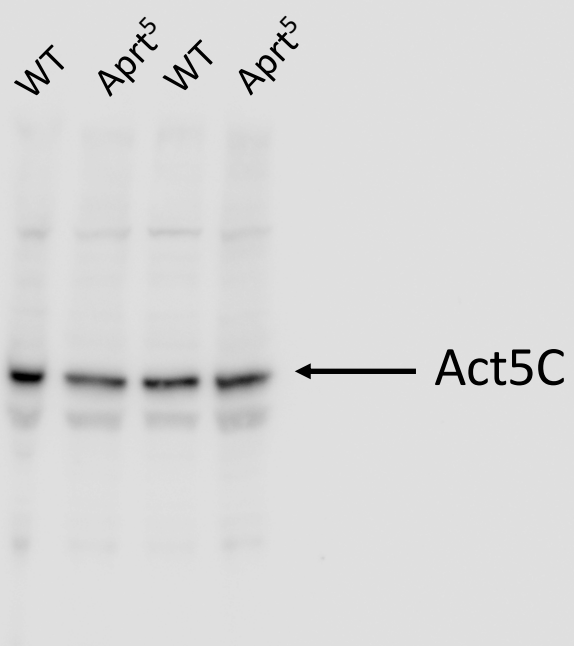

Supplement: Figure 5—source data 3. [file elife-88510-fig5-data3.zip › Figure 5 - source data 3.pdf]

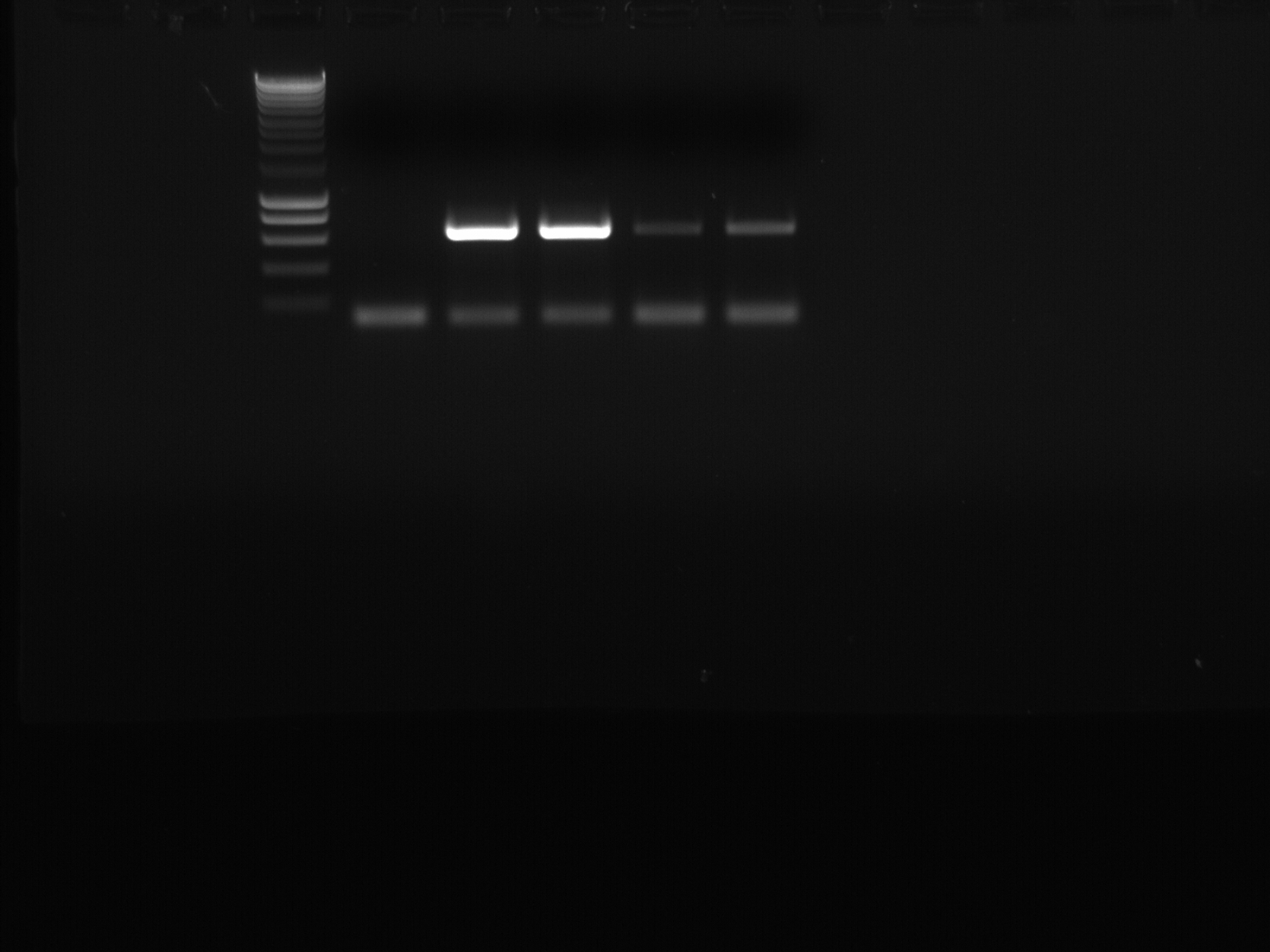

Supplement: Figure 8—source data 1. [file elife-88510-fig8-data1.zip › Figure 8 - source data 1.jpg]

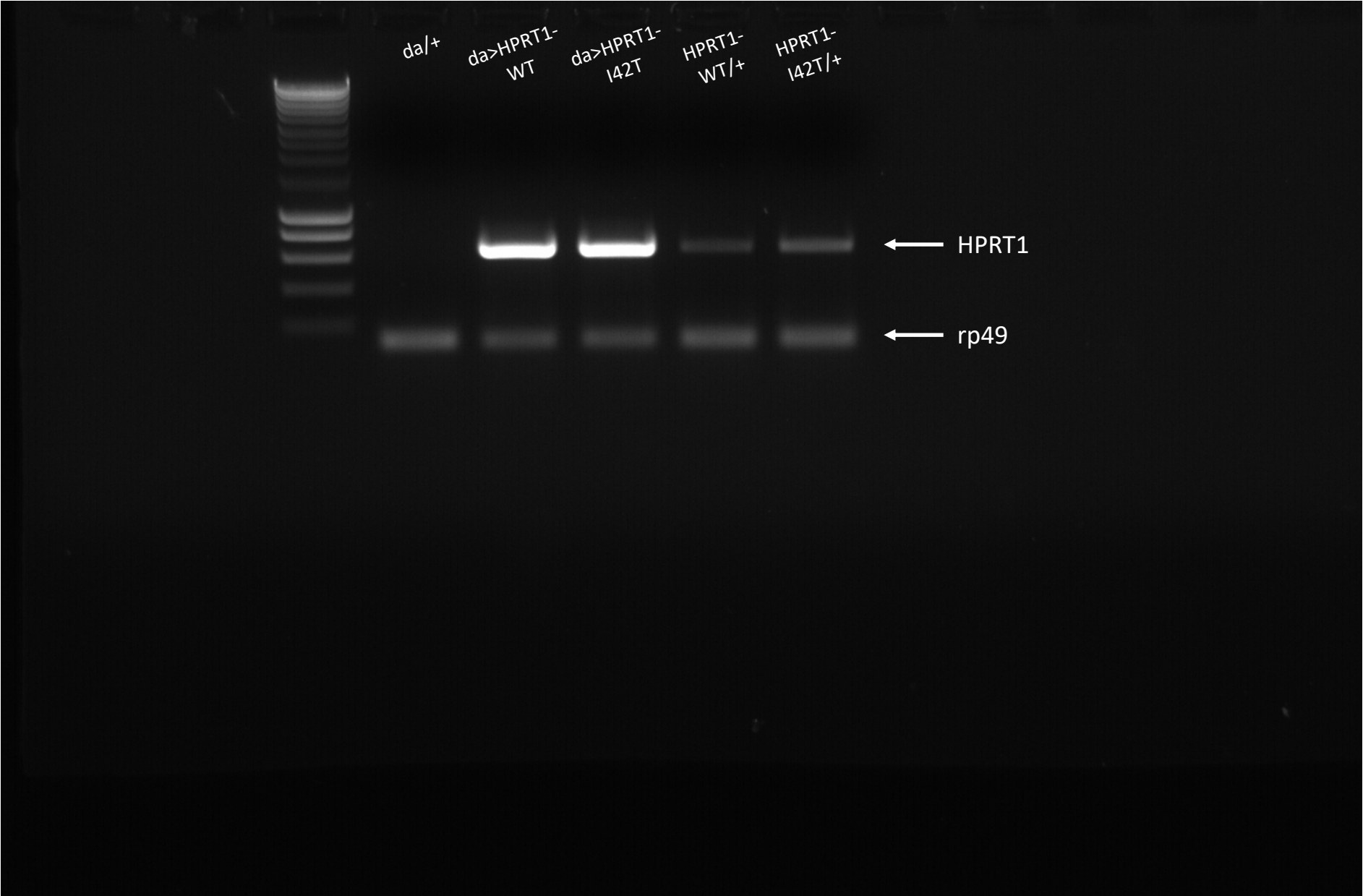

Supplement: Figure 8—source data 2. [file elife-88510-fig8-data2.zip › Figure 8 - source data 2.pdf]
